# Supplementary material for: The rules of multiplayer cooperation in networks of communities
Source: PLoS Comput Biol. 2024 Aug 19;20(8):e1012388. doi: 10.1371/journal.pcbi.1012388 (PMC11361752; doi:10.1371/journal.pcbi.1012388)
Supplement: S1 File — (PDF) [file pcbi.1012388.s001.pdf]

Supplementary material: The rules of multiplayer cooperation in networks of communities

Diogo L. Pires* and Mark Broom†

Department of Mathematics, City, University of London, Northampton Square, London, UK

*Diogo.L.Pires@city.ac.uk

†Mark.Broom@city.ac.uk

August 8, 2024

1 Model Parameters

The general model introduced in the main text of this paper has several free parameters, which we present in Table A. Note that some of the other parameters considered are not free, since they depend on the parameters presented here, e.g. $N = M \times Q$. The results obtained in this work consider different limits of the free parameters. The limit of large home fidelity was considered throughout the whole set of results. In particular, the results presented in section 3.1 of the main text, substantiated by section 2 of the supplementary material, are valid under large home fidelity for arbitrary values of the remaining parameters. In section 3.2, we analyse the expansion of fixation probabilities within the additional limit of weak selection, the results of which are complemented by section 3 of the supplementary material. In sections 3.3 and 3.4, three successive limits are considered: high home fidelity, weak selection and large networks of communities. In section 4 of the supplementary material, we analyse the extent to which these rules are valid outside of the limits of large networks and weak selection.

Overall, we note that some of the limits are interdependent, and that therefore the limit of large home fidelity should be interpreted as being $h/M \to \infty$ and the limit of weak selection as $w \cdot (MQ) \to 0$. The results presented are obtained for general values of community size Q, and general payoff parameters V, K, L and ω.

Notation	Meaning
M	Number of communities
Q	Community size
h	Home fidelity
w	Intensity of selection
V	Social dilemma reward
K	Social dilemma cost
L	Threshold of cooperation
ω	Reward factor used in the PDV

Table A: Free parameters of the used model.

2 Fixation probabilities under high home fidelity

Consider a connected network with M places and an arbitrary set of edges between them. Within the extended territorial raider model, each node will be home to a community of size Q. We consider the limit of large home fidelity, where individuals interact mainly within their communities. This limit is dependent on the size of the network, and therefore when we consider $h \to \infty$, we in fact mean $h/M \to \infty$.

We denote $F^{C_k}_{c,d}$ as the fitness of cooperators C_k in a community with c cooperators and d defectors with home in place P_k. In the limit of high home fidelity, this can be represented as the following expansion:

$$F^{C_k}_{c,d} = 1 - w + w\left[\left(1 - \frac{d_k}{h + d_k}\right)^Q \cdot \prod_{i \in X(k)} \left(1 - \frac{1}{h + d_i}\right)^Q \cdot R^C_{c,d} + \mathcal{O}(h^{-1})\right] = \tag{1}$$
$$= 1 - w + wR^C_{c,d} + \mathcal{O}(h^{-1}),$$

where $X(k)$ denotes the set of places adjacent to P_k on the network, and d_k represents the size of that set, corresponding to the degree of the node. The zeroth-order term of the expansion is dependent only on the composition of the community present in place P_k. Similarly, we have that the fitness of defectors is reduced to the following:

$$F^{D_k}_{c,d} = 1 - w + wR^D_{c,d} + \mathcal{O}(h^{-1}). \quad (2)$$

We denote $f^C_{c,d}$ and $f^D_{c,d}$ as the zeroth-order terms of the fitness expansion under high home fidelity, which are presented in equations 6 and 7 of the main text.

In the next sections, we will focus on the resulting fixation processes in the limit of high home fidelity.

2.1 BDB, DBD, LB and LD dynamics

Starting from the state where all individuals in the population use strategy D, we consider the occurrence of a mutation leading one of them to adopt strategy C. At each step of the BDB process, one individual is chosen for reproduction proportional to their fitness, and another one is chosen for death with probability proportional to the time spent with the first. This means that while there are mixed communities, type-changing replacement events will occur mainly within those communities as we will see below.

We denote r^C_h (r^D_h) as the within-community fixation probability of a single cooperator (defector) in a community of defectors (cooperators). We define this as the probability that starting with one mutant in a community of residents, we will observe the fixation of that mutant in the community before we observe that type vanishing. This probability is equal to the sum of the probabilities of all the paths that alter the number of mutants in that community from 1 to Q without passing by 0. We note that this can be split into the sum of the probabilities of paths under which no type-altering between-community replacements occur before within-community fixation is attained, S_1, S_2, ..., and those under which at least one type-altering between-community replacement occurs before fixation is attained, S'_1, S'_2, ...:

$$r^C_h = p(S_1) + p(S_2) + ... + p(S'_1) + p(S'_2) + ... \quad (3)$$

In the limit we are considering, the sum over the first set of paths introduced before tends to the fixation probability obtained in a well-mixed community [1], since individuals of the same community using the same strategy are indistinguishable. Under the BDB dynamics, the transition probabilities used in this expression can be replaced by the zeroth-order terms of the fitness expansion presented in equations 1 and 2, and higher-order terms are added outside the expression:

$$p(S_1) + p(S_2) + ... = \frac{1}{1 + \sum_{j=1}^{Q-1} \prod_{c=1}^{j} \frac{f^D_{c,Q-c}}{f^C_{c,Q-c}}} + \mathcal{O}(h^{-1}). \quad (4)$$

The paths in the second set introduced above involve at least one between-community replacement, therefore having a probability of at least the first order in h^{-1}. This highlights the fact that they occur at a different time-scale from within-community fixation processes:

$$p(S'_1) + p(S'_2) + ... = \mathcal{O}(h^{-1}). \quad (5)$$

Therefore, the cooperator within-community fixation probability in the limit $h \to \infty$ can be represented as the following:

$$r^C_h = \frac{1}{1 + \sum_{j=1}^{Q-1} \prod_{c=1}^{j} \frac{f^D_{c,Q-c}}{f^C_{c,Q-c}}} + \mathcal{O}(h^{-1}). \quad (6)$$

Similar to this, we can obtain the same equation for the within-community fixation probability of a single defector by using the following expression:

$$r^D_h = \frac{1}{1 + \sum_{j=1}^{Q-1} \prod_{d=1}^{j} \frac{f^C_{Q-d,d}}{f^D_{Q-d,d}}} + \mathcal{O}(h^{-1}). \quad (7)$$

We denote r^C and r^D as the zeroth-order terms of the equations above, which are presented in equations 8 and 8 of the main text.

We call ρ^C the probability that a single mutant cooperator will fixate in a population of defectors. Under $h \to \infty$, populations reach the states where each community is homogeneous, i.e. $c = Q$ or $c = 0$, before any between-community replacement occurs. When the population is in one of the homogeneous community states, it will be altered only when a cooperator replaces a defector from an adjacent community, or vice versa. After a new cooperator (defector) is born, the population will move to a different homogeneous community state with

one more (less) cooperator community with probability r^C (r^D), or it will return to the previous state with probability $1 - r^C$ ($1 - r^D$).

Let us call I the set of communities composed of only cooperators, M the entire set of communities, and $M\backslash I$ the set of communities of defectors. At a homogeneous community state denoted by I, the probability that the size of set I increases by one after a given evolutionary step is:

$$P^{|I|^+}(I) = \left(\frac{f^C_{Q,0}}{|I| \cdot f^C_{Q,0} + |M\backslash I| \cdot f^D_{0,Q}} + \mathcal{O}(h^{-1}) \right) \cdot \left(Q \cdot \sum_{i \in I, j \in M\backslash I} w_{ij} \right) \cdot \left(r^C + \mathcal{O}(h^{-1}) \right). \tag{8}$$

The expression above is the product of probabilities of three successive necessary events: 1) choosing a cooperator from a particular community for birth, 2) choosing a defector from another community to be replaced by the first cooperator, and 3) the within-community fixation of the new cooperator before another between community type-altering event occurs. Note that in the probability of choosing a cooperator for birth, we have replaced the fitness of individuals considered in the homogeneous community state by the zeroth-order terms present in equations 1 and 2, with higher-order terms being explicitly summed onto that probability. The replacement weights w_{ij} between individuals with homes in different places P_i and P_j are independent of their two strategies, and they are multiplied by Q to account for all the defectors present in each of the communities in $M\backslash I$. The within-community fixation probability is perturbed by higher-order terms in h^{-1} already analysed when its expression was obtained. The probability that the size of set I decreases by one is the following:

$$P^{|I|^-}(I) = \left(\frac{f^D_{0,Q}}{|I| \cdot f^C_{Q,0} + |M\backslash I| \cdot f^D_{0,Q}} + \mathcal{O}(h^{-1}) \right) \cdot \left(Q \cdot \sum_{i \in I, j \in M\backslash I} w_{ji} \right) \cdot \left(r^D + \mathcal{O}(h^{-1}) \right). \tag{9}$$

The two equations 8 and 9 depend on the particular set I because the sum of weights w_{ij} depends on it. These weights tend to zero as $h \to \infty$, but they can be considered at their lowest order in h^{-1}. As long as there is no disconnected component of the network of communities ($\forall i \exists j \neq i (w_{ij} \neq 0)$), this probability is low but positive, regardless of the particular set I considered. However, because replacement weights are symmetric, i.e. $w_{ij} = w_{ji}$, the terms in the two transition probabilities in the above equations are identical. Therefore, the ratio between the two probabilities, which we denote Γ, does not depend on the particular set I of communities which are composed of cooperators:

$$\Gamma = \frac{P^{|I|^-}(I)}{P^{|I|^+}(I)} = \frac{f^D_{0,Q} \cdot r^D}{f^C_{Q,0} \cdot r^C}. \tag{10}$$

Furthermore, the transition probability ratio above is constant under all homogeneous community states. After the initial within-community fixation of a cooperator, the probability that the community will fixate on the whole population thus becomes a simple fixed fitness Moran probability [2], with equivalent relative fitness denoted by the ratio from equation 10.

The fixation probability of one single cooperator will therefore be equal to the following:

$$\lim_{h \to \infty} \rho^C = r^C \cdot P_{Moran}\left(\Gamma^{-1}\right) = r^C \cdot \frac{1 - \Gamma}{1 - \Gamma^M}, \tag{11}$$

when $\Gamma \neq 1$. Otherwise, $\lim_{h \to \infty} \rho^C = r^C / M$. Similarly, we have that:

$$\lim_{h \to \infty} \rho^D = r^D \cdot P_{Moran}\left(\Gamma\right) = r^D \cdot \frac{1 - \Gamma^{-1}}{1 - \Gamma^{-M}}, \tag{12}$$

when $\Gamma \neq 1$. Otherwise, $\lim_{h \to \infty} \rho^D = r^D / M$.

This result is surprisingly simple and shows that the topology of the underlying network plays no role in the limit of high home fidelity, as long as there is no disconnected component of the network. The dynamics in that limit depend only on within-community fixation probabilities and on the probability ratio Γ.

The equations presented in 6–12 are valid for dynamics BDB, DBD, LB, and LD. This equivalence is valid under all territorial networks in the limit $h \to \infty$. This is so because the transition probability ratios are the same under all these dynamics, both in each step of the within-community fixation processes considered to build equations 6 and 7, and in the community fixation process as presented in equation 10.

Furthermore, we note that the results are robust to the use of alternative movement models when the limit of isolated communities with the same size is considered, and if the replacement weights between any two individuals are kept symmetrical, i.e. the evolutionary graph is undirected. This includes simple variations of the current movement model, e.g. all individuals could have the same probability of not being in their home node independent of its degree, in the limit where this probability tends to zero. Even though outside the limit of isolated communities, those models could lead to generally different results, in that limit the fixation probabilities would be equal to the ones obtained here. Nonetheless, these alternative movement model choices could still impact the rate at which between-community events occur.

2.2 Failure of fixation of cooperators under the CPD with BDB dynamics

Under the CPD with BDB, the effective fitness Γ of the between-community process can be obtained using equation 10. Replacing r^D/r^C with the simplified ratio between the two probabilities [3, 4], we obtain the following explicit simplified expression for Γ:

$$\Gamma = \frac{f^D_{0,Q}}{f^C_{Q,0}} \cdot \prod_{c=1}^{Q-1} \frac{f^D_{c,Q-c}}{f^C_{c,Q-c}}. \tag{13}$$

We split the denominator and numerator of the previous product into two products and apply the definition of rewards under the CPD (see Table 1 of the main text), thus obtaining the following:

$$\Gamma = \frac{1-w}{1-w+w(V-K)} \frac{\prod_{c=1}^{Q-1} 1-w+w\dfrac{c}{Q-1}V}{\prod_{c'=1}^{Q-1} 1-w+w\left(\dfrac{c'-1}{Q-1}V-K\right)}. \tag{14}$$

We note that an extension of the products in the numerator and denominator to $c=0$ and $c'=Q$ respectively, would include the extra terms multiplied by each of the products. Doing that, together with the change of variable $c=c'-1$, we obtain the following:

$$\begin{aligned}\Gamma &= \frac{\prod_{c=0}^{Q-1} 1-w+w\dfrac{c}{Q-1}V}{\prod_{c'=1}^{Q} 1-w+w\left(\dfrac{c'-1}{Q-1}V-K\right)} = \frac{\prod_{c=0}^{Q-1} 1-w+w\dfrac{c}{Q-1}V}{\prod_{c=0}^{Q-1} 1-w+w\left(\dfrac{c}{Q-1}V-K\right)} = \\ &= \prod_{c=0}^{Q-1} \frac{1-w+w\dfrac{c}{Q-1}V}{1-w+w\left(\dfrac{c}{Q-1}V-K\right)}.\end{aligned} \tag{15}$$

We have that $\Gamma > 1$ for any choice of payoff parameters, intensity of selection, and community size. This means that the Moran probability will always be lower than $1/M$. At the same time, we note that $r^C < 1/Q$ because under the CPD, cooperators always do worse than defectors in the same group. Therefore, when we consider the BDB dynamics under the CPD with high home fidelity, cooperators never fixate above the neutral probability $1/(MQ)$ for any community number and size, network topology, and payoff parameter choices.

2.3 DBB and BDD dynamics

A procedure analogous to the one conducted in section 2.1 applies to the remaining two dynamics presented in this paper. However, these dynamics exhibit distinct transition probability ratios compared to the four aforementioned ones, resulting in quantitatively different outcomes.

We start by noting that the sum of paths that end in fixation with no type-altering between-community replacements is obtained using different transition probabilities. Let us start with the DBB dynamics, under which the transition probability from having c cooperators to having $c+1$ or $c-1$ at a given evolutionary step are respectively as follows:

$$P^+_{DBB}(c, Q-c) = \frac{Q-c}{N} \cdot \frac{c \cdot f^C_{c,Q-c}}{c \cdot f^C_{c,Q-c} + (Q-c-1) \cdot f^D_{c,Q-c}} + \mathcal{O}(h^{-1}), \tag{16}$$

$$P^-_{DBB}(c, Q-c) = \frac{c}{N} \frac{(Q-c) \cdot f^D_{c,Q-c}}{(c-1) \cdot f^C_{c,Q-c} + (Q-c) \cdot f^D_{c,Q-c}} + \mathcal{O}(h^{-1}). \tag{17}$$

Repeating this process considering the BDD dynamics, we obtain the following transition probabilities, which were simplified by multiplying the numerator and denominator by both cooperator and defector's fitness:

$$\begin{aligned}P^+_{BDD}(c, Q-c) &= \frac{c}{N} \cdot \frac{(Q-c) \cdot \left(f^D_{c,Q-c}\right)^{-1}}{(Q-c) \cdot \left(f^D_{c,Q-c}\right)^{-1} + (c-1) \cdot \left(f^C_{c,Q-c}\right)^{-1}} + \mathcal{O}(h^{-1}) = \\ &= \frac{c}{N} \cdot \frac{(Q-c) \cdot f^C_{c,Q-c}}{(Q-c) \cdot f^C_{c,Q-c} + (c-1) \cdot f^D_{c,Q-c}} + \mathcal{O}(h^{-1}),\end{aligned} \tag{18}$$

$$P^{-}_{BDD}(c,Q-c)=\frac{Q-c}{N}\cdot\frac{c\cdot\left(f^{C}_{c,Q-c}\right)^{-1}}{(Q-c-1)\cdot\left(f^{D}_{c,Q-c}\right)^{-1}+c\cdot\left(f^{C}_{c,Q-c}\right)^{-1}}+\mathcal{O}(h^{-1})=$$
$$=\frac{Q-c}{N}\cdot\frac{c\cdot f^{D}_{c,Q-c}}{(Q-c-1)\cdot f^{C}_{c,Q-c}+c\cdot f^{D}_{c,Q-c}}+\mathcal{O}(h^{-1}). \tag{19}$$

The ratio $U(c,d)=P^{-}(c,d)/P^{+}(c,d)$ between transition probabilities under both dynamics leads to the following expression:

$$U_{DBB/BDD}(c,Q-c)=\frac{P^{-}_{DBB/BDD}(c,Q-c)}{P^{+}_{DBB/BDD}(c,Q-c)}=$$
$$=\frac{f^{D}_{c,Q-c}}{f^{C}_{c,Q-c}}\cdot\left(\frac{T_{DBB/BDD}(c,Q-c)-f^{D}_{c,Q-c}}{T_{DBB/BDD}(c,Q-c)-f^{C}_{c,Q-c}}\right)= \tag{20}$$
$$=\frac{f^{D}_{c,Q-c}}{f^{C}_{c,Q-c}}\left(1+\frac{f^{C}_{c,Q-c}-f^{D}_{c,Q-c}}{T_{DBB/BDD}(c,Q-c)-f^{C}_{c,Q-c}}\right),$$

where we have used the following definitions:

$$T_{DBB}(c,d)=c\cdot f^{C}_{c,d}+d\cdot f^{D}_{c,d}, \tag{21}$$

$$T_{BDD}(c,d)=d\cdot f^{C}_{c,d}+c\cdot f^{D}_{c,d}. \tag{22}$$

Now, we use these transition probability ratios to compute the zeroth-order term of the within-community fixation probability expansion, similar to what was done in section 2.1, getting the following result:

$$r^{C,h}_{DBB/BDD}=\frac{1}{1+\sum_{j=1}^{Q-1}\prod_{c=1}^{j}\frac{f^{D}_{c,Q-c}}{f^{C}_{c,Q-c}}\left(1+\frac{f^{C}_{c,Q-c}-f^{D}_{c,Q-c}}{T_{DBB/BDD}(c,Q-c)-f^{C}_{c,Q-c}}\right)}+\mathcal{O}(h^{-1}). \tag{23}$$

Following the same procedure for the within-community fixation of defectors, we get the following result:

$$r^{D,h}_{DBB/BDD}=\frac{1}{1+\sum_{j=1}^{Q-1}\prod_{d=1}^{j}\frac{f^{C}_{Q-d,d}}{f^{D}_{Q-d,d}}\cdot\left(1+\frac{f^{D}_{Q-d,d}-f^{C}_{Q-d,d}}{T_{DBB/BDD}(Q-d,d)-f^{D}_{Q-d,d}}\right)}+\mathcal{O}(h^{-1}). \tag{24}$$

We denote $r^{C}_{DBB/BDD}$ and $r^{D}_{DBB/BDD}$ as the zeroth-order terms of the equations above, which are presented in equations 15 and 16 of the main text.

The difference in transition probabilities, when compared to the previous 4 dynamics, also affects the probability that the number of communities increases or decreases by one in the next evolutionary step. We start by looking at what happens under the DBB dynamics:

$$P^{|I|^{+}}_{DBB}(I)=\left(\frac{1}{M}+\mathcal{O}(h^{-1})\right)\cdot\left(Q\cdot\left(\sum_{i\in I,j\in M\setminus I}w_{ij}\right)\cdot\left(\frac{f^{C}_{Q,0}}{f^{D}_{0,Q}}+\mathcal{O}(h^{-1})\right)\right)\cdot\left(r^{C}_{DBB}+\mathcal{O}(h^{-1})\right). \tag{25}$$

The preceding probability encompasses: 1) the uniform random selection of a specific community for the death of one of its individuals; 2) the subsequent selection, if the first individual was a defector, of a cooperator community for birth, involving any of its Q cooperators; and 3) the fixation of the invading cooperator in the newly mixed community. The sum of fractions above includes a simplification coming from the fact that the denominator is a sum over all products of weights and fitness according to the definition from Table 2 of the main text, which in the limit $h\to\infty$ simply tends to the fitness of communal residents $f^{D}_{0,Q}$ plus higher-order terms in h^{-1}. This will introduce another key difference in the results. We obtain the following transition probability in the opposing direction:

$$P^{|I|^{-}}_{DBB}(I)=\left(\frac{1}{M}+\mathcal{O}(h^{-1})\right)\cdot\left(Q\cdot\left(\sum_{i\in I,j\in M\setminus I}w_{ji}\right)\cdot\left(\frac{f^{D}_{0,Q}}{f^{C}_{Q,0}}+\mathcal{O}(h^{-1})\right)\right)\cdot\left(r^{D}_{DBB}+\mathcal{O}(h^{-1})\right). \tag{26}$$

Now looking at what happens under BDD dynamics, we obtain the following expressions for transition probabilities between homogeneous community states:

$$P^{|I|^{+}}_{BDD}(I)=\left(\frac{1}{M}+\mathcal{O}(h^{-1})\right)\cdot\left(Q\cdot\left(\sum_{i\in I,j\in M\setminus I}w_{ij}\right)\cdot\left(\frac{\left(f^{D}_{0,Q}\right)^{-1}}{\left(f^{C}_{Q,0}\right)^{-1}}+\mathcal{O}(h^{-1})\right)\right)\cdot\left(r^{C}_{BDD}+\mathcal{O}(h^{-1})\right), \tag{27}$$

$$P_{BDD}^{|I|^-}(I) = \left(\frac{1}{M} + \mathcal{O}(h^{-1})\right) \cdot \left(Q \cdot \left(\sum_{i \in I, j \in M \setminus I} w_{ij}\right) \cdot \left(\frac{\left(f_{Q,0}^C\right)^{-1}}{\left(f_{0,Q}^D\right)^{-1}} + \mathcal{O}(h^{-1})\right)\right) \cdot \left(r_{BDD}^C + \mathcal{O}(h^{-1})\right). \quad (28)$$

We note that, once again, the highest-order terms in these probabilities are indeed first-order in h^{-1} due to the effects of between-community replacements happening between different communities on the network. However, the particular set of edges between the nodes of the network, i.e. its topology, does not influence the ratio between probabilities, but only the time-scale at which these transitions occur. The probability ratio $\Gamma_{DBB/BDD}$ is independent of I and its size, as was under the remaining dynamics:

$$\Gamma_{DBB/BDD} = \frac{P_{DBB/BDD}^{|I|^-}(I)}{P_{DBB/BDD}^{|I|^+}(I)} = \left(\frac{f_{0,Q}^D}{f_{Q,0}^C}\right)^2 \cdot \frac{r_{DBB/BDD}^D}{r_{DBB/BDD}^C}. \quad (29)$$

Therefore the resulting process under high home fidelity in these two dynamics is parallel to the one occurring under the remaining four dynamics, with two quantitative differences: within-community fixation probabilities have correction coefficients as represented in equations 23 and 24, and the overall population process has an altered equivalent fitness characterised in equation 29.

The resulting fixation probabilities are therefore the following:

$$\lim_{h \to \infty} \rho_{DBB/BDD}^C = r_{DBB/BDD}^C \cdot P_{Moran}\left(\Gamma_{DBB/BDD}^{-1}\right) = r_{DBB/BDD}^C \cdot \frac{1 - \Gamma_{DBB/BDD}}{1 - \Gamma_{DBB/BDD}^M}, \quad (30)$$

when $\Gamma_{DBB/BDD} \neq 1$. Otherwise, $\lim_{h \to \infty} \rho_{DBB/BDD}^C = r_{DBB/BDD}^C / M$. Similarly, we have that:

$$\lim_{h \to \infty} \rho_{DBB/BDD}^D = r_{DBB/BDD}^D \cdot P_{Moran}\left(\Gamma_{DBB/BDD}\right) = r_{DBB/BDD}^D \cdot \frac{1 - \Gamma_{DBB/BDD}^{-1}}{1 - \Gamma_{DBB/BDD}^{-M}}, \quad (31)$$

when $\Gamma_{DBB/BDD} \neq 1$. Otherwise, $\lim_{h \to \infty} \rho_{DBB/BDD}^D = r_{DBB/BDD}^D / M$.

3 Fixation probabilities under high home fidelity and weak selection

Making the assumption of high home fidelity, we now introduce the limit of weak selection. Both limits considered depend on the number of places on the network, since large home fidelity in fact means $h/M \to \infty$ and weak selection means to $w \cdot (MQ) \to 0$. Therefore, when large networks are further considered, the values of h and w have to be chosen accordingly. We highlight the fact that the limits are considered in this order: first, we consider home fidelity to be asymptotically high, then we consider selection to be asymptotically weak, and only then may we consider large networks. It has been proved that the order in which the limits of weak selection and large population size are considered impacts the resulting asymptotic fixation probability expansions and the conditions for the evolution of a given strategy to be favoured in comparison to neutral fixation [4].

3.1 BDB, DBD, LB, and LD dynamics

We start from equation 11 and expand it around $w \to 0$. In that case, we obtain the following expression:

$$\rho^C \approx \left.\left[r^C \cdot \frac{1 - \Gamma}{1 - \Gamma^M}\right]\right|_{w \to 0} + w \left.\left[\frac{\partial}{\partial w}\left(\frac{1 - \Gamma}{1 - \Gamma^M}\right) \cdot r^C + \frac{\partial r^C}{\partial w} \cdot \left(\frac{1 - \Gamma}{1 - \Gamma^M}\right)\right]\right|_{w \to 0}. \quad (32)$$

We start to simplify this equation by noting that, under this limit, within-community fixation probabilities tend to $1/Q$. In that limit, we define their derivatives in respect to w as the following:

$$\delta^C = \left.\frac{\partial r^C}{\partial w}\right|_{w \to 0} = \frac{1}{Q^2} \sum_{j=1}^{Q-1} \sum_{c=1}^{j} [R_{c,Q-c}^C - R_{c,Q-c}^D], \quad (33)$$

$$\delta^D = \left.\frac{\partial r^D}{\partial w}\right|_{w \to 0} = \frac{1}{Q^2} \sum_{j=1}^{Q-1} \sum_{d=1}^{j} \left[R_{Q-d,d}^D - R_{Q-d,d}^C\right]. \quad (34)$$

.

These equations can be simplified by taking into account that each term on the inner sum is repeated $Q-c$ and $Q-d$ times respectively in the outer sum, thus leading to the following expressions:

$$\delta^C = \frac{1}{Q^2}\sum_{c=1}^{Q-1}(Q-c)[R^C_{c,Q-c} - R^D_{c,Q-c}], \tag{35}$$

$$\delta^D = \frac{1}{Q^2}\sum_{d=1}^{Q-1}(Q-d)\left[R^D_{Q-d,d} - R^C_{Q-d,d}\right]. \tag{36}$$

.

In the same limit, we observe that $\Gamma \to 1$, leading the Moran probability with effective fitness Γ to simply tend to $1/M$. We then evaluate the derivative of the Moran probability and obtain the following relation:

$$\frac{\partial}{\partial w}\left(\frac{1-\Gamma}{1-\Gamma^M}\right)\bigg|_{w\to 0} = \frac{1}{2}\left(1-\frac{1}{M}\right)\left(-\frac{\partial \Gamma}{\partial w}\bigg|_{w\to 0}\right). \tag{37}$$

The derivative of the effective fitness Γ can be obtained in the following way:

$$-\frac{\partial \Gamma}{\partial w}\bigg|_{w\to 0} = \Delta^{CD} + Q\left(\delta^C - \delta^D\right), \tag{38}$$

where

$$\Delta^{CD} = R^C_{Q,0} - R^D_{0,Q} = -\Delta^{DC}. \tag{39}$$

Replacing these redefined terms onto the original equation 32 of the expanded fixation probability, we obtained the following relation:

$$\rho^C \approx \frac{1}{MQ} + \frac{w}{2}\left[\frac{1}{Q}\left(1-\frac{1}{M}\right)\Delta^{CD} + \left(1+\frac{1}{M}\right)\delta^C - \left(1-\frac{1}{M}\right)\delta^D\right]. \tag{40}$$

Following the same procedure for the fixation probabilities of defectors, we obtain the previous equation with swapped indexes C and D:

$$\rho^D \approx \frac{1}{MQ} + \frac{w}{2}\left[\frac{1}{Q}\left(1-\frac{1}{M}\right)\Delta^{DC} + \left(1+\frac{1}{M}\right)\delta^D - \left(1-\frac{1}{M}\right)\delta^C\right]. \tag{41}$$

3.2 DBB and BDD dynamics

The expansion is slightly different when we consider the DBB and BDD dynamics. The original expansion is parallel to the one presented in equation 32, the only difference being that all instances of r^C, r^D, and Γ are replaced by their respective equations under the DBB and BDD dynamics. Evaluated in the limit $w \to 0$, the three quantities lead to the same values as in the previous dynamics. Therefore, all differences come from their derivatives. Based on the definitions presented in equations 23 and 24, and the previously defined derivatives δ^C and δ^D, we obtain the following relations for their derivatives evaluated in the limit $w \to 0$:

$$\begin{aligned}\frac{\partial r^C_{DBB}}{\partial w}\bigg|_{w\to 0} = \frac{\partial r^C_{BDD}}{\partial w}\bigg|_{w\to 0} &= \frac{1}{Q^2}\sum_{j=1}^{Q-1}\sum_{c=1}^{j}\left(1-\frac{1}{Q-1}\right)[R^C_{c,Q-c} - R^D_{c,Q-c}] \\ &= \left(1-\frac{1}{Q-1}\right)\delta^C,\end{aligned} \tag{42}$$

$$\begin{aligned}\frac{\partial r^D_{DBB}}{\partial w}\bigg|_{w\to 0} = \frac{\partial r^D_{BDD}}{\partial w}\bigg|_{w\to 0} &= \frac{1}{Q^2}\sum_{j=1}^{Q-1}\sum_{d=1}^{j}\left(1-\frac{1}{Q-1}\right)\left[R^D_{Q-d,d} - R^C_{Q-d,d}\right] \\ &= \left(1-\frac{1}{Q-1}\right)\delta^D.\end{aligned} \tag{43}$$

The derivative of the effective fitness Γ can be obtained in the following way:

$$\frac{\partial \Gamma_{DBB/BDD}}{\partial w}\bigg|_{w\to 0} = 2\left[R^D_{0,Q} - R^C_{Q,0}\right] + Q\left(\frac{\partial r^D_{DBB/BDD}}{\partial w}\bigg|_{w\to 0} - \frac{\partial r^C_{DBB/BDD}}{\partial w}\bigg|_{w\to 0}\right), \tag{44}$$

which, based on equations 39, 42 and 43, leads to the following equation:

$$-\frac{\partial \Gamma_{DBB/BDD}}{\partial w}\bigg|_{w\to 0} = 2\Delta^{CD} + Q\left(1-\frac{1}{Q-1}\right)\left(\delta^C - \delta^D\right). \tag{45}$$

Replacing these terms in the fixation probability expansion parallel to the one from equation 32, we obtain the following resulting equations:

$$\begin{aligned}\rho^{C}_{DBB/BDD} \approx \frac{1}{MQ} + \frac{w}{2}\Bigg[&2\frac{1}{Q}\left(1-\frac{1}{M}\right)\Delta^{CD} + \\ &+\left(1-\frac{1}{Q-1}\right)\left(1+\frac{1}{M}\right)\delta^{C} - \left(1-\frac{1}{Q-1}\right)\left(1-\frac{1}{M}\right)\delta^{D}\Bigg],\end{aligned} \tag{46}$$

$$\begin{aligned}\rho^{D}_{DBB/BDD} \approx \frac{1}{MQ} + \frac{w}{2}\Bigg[&2\frac{1}{Q}\left(1-\frac{1}{M}\right)\Delta^{DC} + \\ &+\left(1-\frac{1}{Q-1}\right)\left(1+\frac{1}{M}\right)\delta^{D} - \left(1-\frac{1}{Q-1}\right)\left(1-\frac{1}{M}\right)\delta^{C}\Bigg].\end{aligned} \tag{47}$$

3.3 Obtaining the rules of cooperation for general social dilemmas

To achieve the resulting rules presented in section 3.2 of the main text, we systematically compute the terms Δ^{CD}, δ^{C} and δ^{D} under each of the general social dilemmas approached. These are summarised in the following table:

Multiplayer Game	Δ^{CD}	δ^{C}	δ^{D}
CPD	$V-K$	$-\frac{Q-1}{2Q}\left(K+\frac{V}{Q-1}\right)$	$\frac{Q-1}{2Q}\left(K+\frac{V}{Q-1}\right)$
PD, VD	$V-K$	$-\frac{Q-1}{2Q}K$	$\frac{Q-1}{2Q}K$
PDV	$\frac{V}{Q}\frac{1-\omega^{Q}}{1-\omega}-K$	$-\frac{Q-1}{2Q}K$	$\frac{Q-1}{2Q}K$
S	$V-K/Q$	$-\frac{1}{Q}\left(H_Q-1\right)K$	$\frac{Q-1}{Q^2}K$
TVD, SH	$\begin{cases}V-K & Q\geq L\\ -K & Q<L\end{cases}$	$-\frac{Q-1}{2Q}K$	$\frac{Q-1}{2Q}K$
FSH	$\begin{cases}\frac{V}{Q}-K & Q\geq L\\ -K & Q<L\end{cases}$	$-\frac{Q-1}{2Q}K$	$\frac{Q-1}{2Q}K$
TS	$\begin{cases}V-K/Q & Q\geq L\\ -K/L & Q<L\end{cases}$	$\begin{cases}-\frac{K}{Q^2}\left(Q(H_{Q-1}-H_L)+\frac{L+1}{2}\right) & Q\geq L\\ -\frac{Q-1}{2Q}\frac{K}{L} & Q<L\end{cases}$	$\begin{cases}\frac{1}{Q^2}\frac{K}{2}(2Q-L-1) & Q\geq L\\ \frac{Q-1}{2Q}\frac{K}{L} & Q<L\end{cases}$
HD	$\frac{Q-1}{Q}K$	$\frac{Q-1}{Q^2}\left(\left(\frac{Q}{2}-1\right)K-V\right)$	$\frac{1}{Q}\left(\left(H_Q-1\right)V-\left(\frac{Q+1}{2}-H_Q\right)K\right)$

Table B: Value of fixation probability expansion terms under weak selection for each social dilemma. The terms denote the contributions of between-community events (Δ^{CD}), within-community fixation of cooperators (δ^{C}) and defectors (δ^{D}). Their definitions can be found in equations 35, 36, and 39.

The values of Δ^{CD} can be trivially obtained based on the calculation of the rewards among communal cooperators and communal defectors. The values of δ^{C} and δ^{D} are often simple to calculate because payoff differences between cooperators and defectors in mixed communities are constant under most social dilemmas. The only dilemmas under which this is not as trivial are the S, the TS and the HD dilemmas, under which we had to include the harmonic series defined as the following:

$$H_Q = \sum_{i=1} \frac{1}{i}. \tag{48}$$

4 Rules of cooperation under a finite number of communities and general intensity of selection

In this section, we propose to analyse the evolution and stability of cooperation when relaxing the limits of weak selection (considered in sections 3.2–3.4 of the main text) and large number of communities (considered in sections 3.3 and 3.4 of the main text). We start by considering a finite number of communities under weak selection and their impact on the simple rules previously presented. We derive an exact rule for the CPD and analyse the general impact of finiteness under the remaining social dilemmas. We then move outside the weak selection limit, analysing the impact of relaxing the two limits on the parameter regions under which cooperation evolves.

4.1 The effect of a finite number of communities on the evolution of cooperation

We start by analysing the particular case of the CPD under the DBB and BDD dynamics. The fixation probability of cooperators expanded under weak selection is larger than the neutral value if the following condition is true:

$$V/K > (Q-1)\cdot\frac{1-\frac{2}{MQ}}{1-\frac{2(Q-1)}{MQ}}. \tag{49}$$

The second term of the product on the right-hand side of the equation can be considered the finiteness correction coefficient. This is equal to 1 under $Q = 2$, which means that in that case, the condition obtained is the same regardless of the number of communities. However, for larger numbers of communities ($Q > 2$), the denominator is lower than the numerator in the correction coefficient above, and therefore the critical value of the reward-to-cost ratio will necessarily be larger than the one obtained under an infinite number of communities. The difference between the two should be the largest for the smallest possible network size $M = 2$, under which the rule becomes the following:

$$V/K > (Q-1)\cdot(Q-1). \tag{50}$$

It was stated in section 3.2 of the main text that decreasing the number of communities increases the importance of within-community fixation against between-community replacement events in the course of a fixation process. Because of that, defectors should generally do better in smaller networks. This can be concluded based on the following rearrangement of the weak selection expansion:

$$\begin{aligned}\rho^C \approx \frac{1}{MQ}+\frac{w}{2}\Bigg[2\frac{1}{Q}\Delta^{CD}+&\left(1-\frac{1}{Q-1}\right)(\delta^C-\delta^D)+\\ &+\frac{1}{M}\left[-\frac{2}{Q}\Delta^{CD}+\left(1-\frac{1}{Q-1}\right)\left(\delta^C+\delta^D\right)\right]\Bigg].\end{aligned} \tag{51}$$

We can identify three types of terms in the equation above. The first type corresponds to the zeroth order term of the fixation probability under weak selection; the second includes the set of first-order terms in w which are independent of M; and the third (second line of the equation) represents the first-order terms in w which are dependent on M and vanish for large M, thus having a finiteness correction of the expansion. The third type does not originate on the expansion of the fixation probability under a large number of communities but instead reflects its *exact* dependence on the number of communities under weak selection. Let us analyse the effect introduced by this finiteness correction term.

Focusing on public goods dilemmas, the fixation of cooperators can only be favoured by selection for a choice of network parameters Q and M if we observe $\Delta^{CD} > 0$. This is so because the remaining contributions in equations 40 and 46 (involving δ^C and $-\delta^D$) are always negative (see Table B). Therefore, for cooperation to fixate successfully, the first contribution to the finiteness correction in equation 51 has to be negative. The sum of the remaining correction contributions is zero in most public goods dilemmas, except for the S and the TS dilemmas, under which they are negative. This means that the M-dependent term of the weak selection expansion of the fixation probability is necessarily negative. If the fixation probability is higher than the neutral one for a given choice of network (Q and M) and payoff (V and K) parameters, it will necessarily be so for any M larger than that, whereas it might not be for choices of M lower than that. This necessarily means that the critical reward-to-cost ratio under all public goods dilemmas (expressed in Table 3 of the main text for a large number of communities) will increase when we decrease M. Finiteness narrows the regions of V/K for which cooperation evolves under public goods dilemmas.

Under the HD dilemma, the effect of M can be quite different because both Δ^{CD} and $\delta^C + \delta^D$ are always positive, thus leading to different signs on the two contributions to the third term in the previous equation. This complex effect of M is parallel to the effects of Q explored in section 3.2 of the main text.

Cooperation evolves under sufficiently large values of V/K when $Q \geq 2$ in non-threshold public goods and when $Q \geq L$ in threshold public goods, irrespective of the number of communities M. This conclusion arises from the linear dependency of all Δ^{CD} on V, whereas δ^C and δ^D lack such dependence except under the CPD (a game already shown to support cooperation under any number of communities through equation 49). Consequently, there is always a critical value of V above which the first-order term of the weak selection expansion is positive. In the context of the HD dilemma, cooperation can consistently evolve under sufficiently small values of V/K when $Q \geq 2$, regardless of the network size. This stems from the linear dependence of Δ^{CD} and δ^C on K, and δ^D on $-K$, ensuring that all contributions to the fixation probability expansion are positive when K reaches a high enough value.

4.2 The effect of strong selection on the evolution of cooperation

In this section, we relax the weak selection limit. This limit was introduced in section 3.2 of the main text, and it was used together with the limit of large number of communities in the succeeding sections to achieve simple

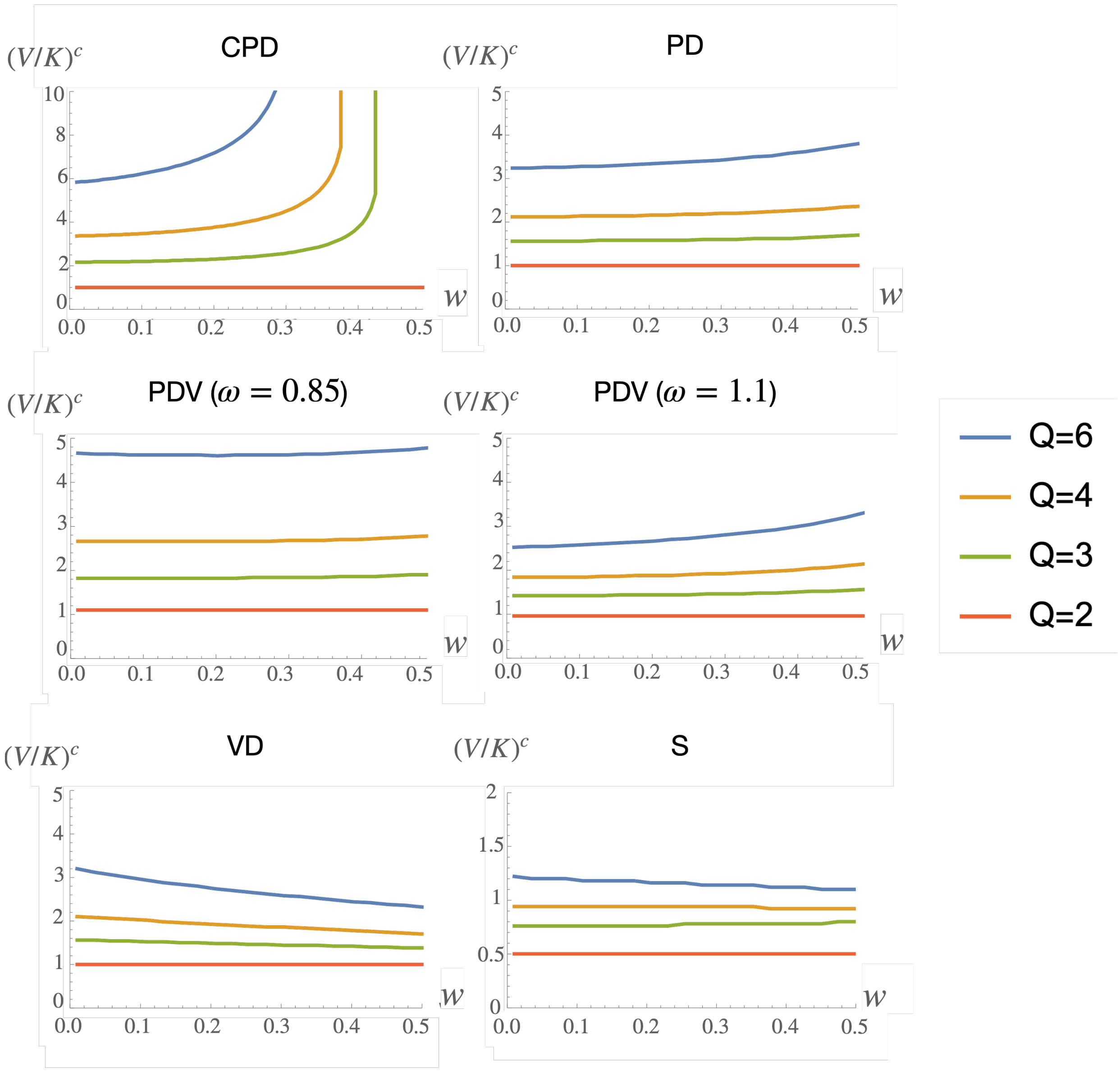

Figure A: Critical values of the reward-to-cost ratio for which $V/K > (V/K)^c$ leads to the successful fixation and stability of cooperation. The results are obtained for different intensities of selection under $M = 10$, $K = 1$, and different values of Q.

rules of cooperation. We focus on understanding the effect of considering larger values of intensity of selection on the critical value of the reward-to-cost ratio, denoted as $(V/K)^c$, above which cooperation fixates in public goods dilemmas and below which cooperation fixates in the HD dilemma. Figs A, B, and C show the value of $(V/K)^c$ under networks with $M = 10$ communities of various sizes, such as $Q = 2, 3, 4, 6, 8$. We present results for values of w between 0 and 0.5, the interval under which all 10 social dilemmas can be considered for any possible value of V when $K = 1$, i.e. for which probabilities remain positive. More generally, this corresponds to $w \in (0, 1/(K + 1))$.

The reward-to-cost ratio is affected in different ways by the increase in intensity of selection for each of the social dilemmas, and no particular strategy is consistently favoured. We start by focusing on the CPD. Under $Q = 2$, cooperation evolves for $V/K > 1$ for all values of w and M, which is equivalent to the rule shown in Table 3 of the main text. However, under the remaining community sizes considered, increasing the intensity of selection consistently leads to higher critical values of the reward-to-cost ratio. In those cases, weak selection has a positive effect on the evolution of cooperation. Under strong enough selection, there is no critical reward-to-cost ratio and cooperation may never evolve. We explored other values of M and observed that increasing M under strong selection extended the values of w for which there existed a critical reward-to-cost ratio.

Under other dilemmas such as the PD, the PDV, and the TS, lower intensities of selection also led to lower critical values of the reward-to-cost ratio, but the differences obtained across values of w are substantially lower. In those dilemmas, the payoff parameters we explored always led to the existence of a critical value, contrary to

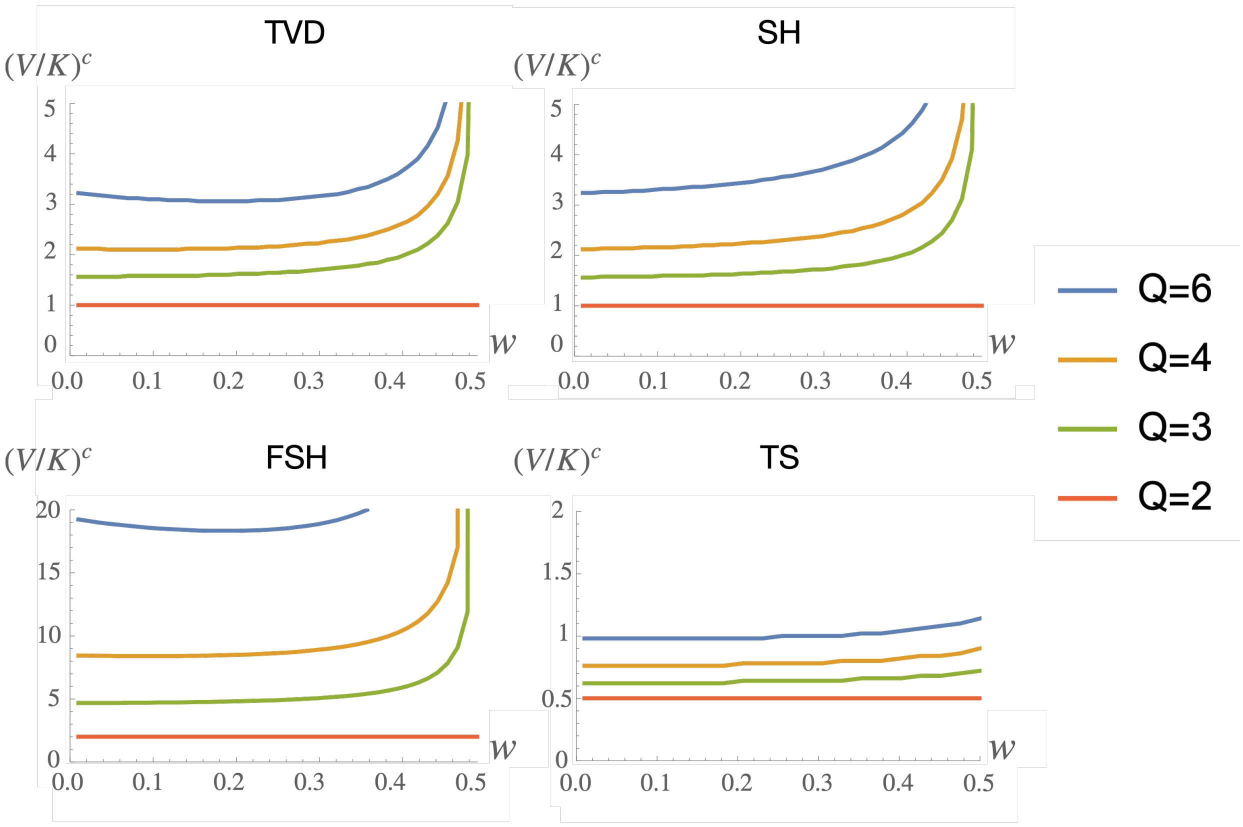

Figure B: Critical values of the reward-to-cost ratio for which $V/K > (V/K)^c$ leads to the successful fixation and stability of cooperation. The results are obtained for different intensities of selection under $M = 10$, $K = 1$, $L = 2$, and different values of Q.

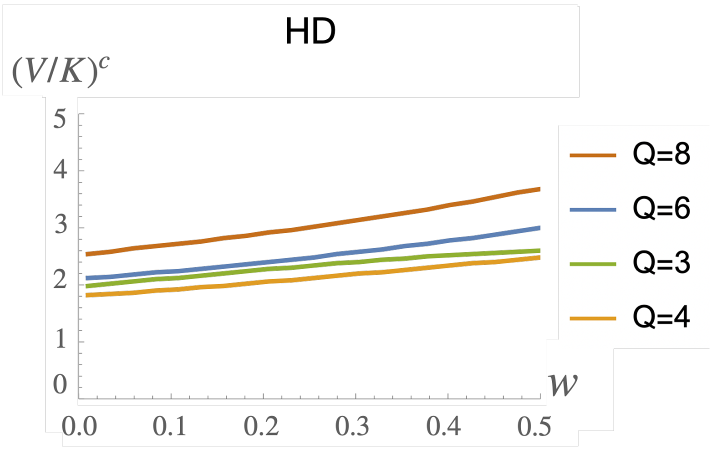

Figure C: Critical values of the reward-to-cost ratio for which $V/K < (V/K)^c$ leads to the successful fixation of cooperation. Cooperators fixate for all values of V/K and w when $Q = 2$. The results are obtained for different intensities of selection under $M = 10$, $K = 1$, $L = 2$, and different values of Q.

what was observed under the CPD and other threshold games such as the TVD, SH and the FSH, which showed otherwise similar trends. Moreover, under the PDV, the TVD, and the FSH, the minimum value of $(V/K)^c$ occurred for intermediate intensities of selection. These correspond to an optimal w for which cooperation evolves under the largest regions of the payoff parameter space.

Under the VD and the S, higher intensities of selection lead to lower critical values of the reward-to-cost ratio. Therefore, cooperation evolves for larger regions of the payoff parameter space under stronger selection. This trend was more pronounced under the VD.

As an overall trend under public goods dilemmas, we note that larger community sizes require higher reward-to-cost ratios for cooperators to successfully fixate. This is concluded from the rules of multiplayer cooperation (section 3.3 of the main text), obtained under weak selection and a large number of communities. Here, we observe that this is still valid when those limits are relaxed.

Furthermore, we can observe some of the effects of considering a finite number of communities. The critical values obtained under $w \to 0$ and shown in Figs A and B for public goods dilemmas are higher than the ones presented in Table 3 of the main text, which were obtained considering a large number of communities. Larger numbers of communities were proven in section 4.1 to decrease the values of $(V/K)^c$ above which cooperation evolves under public goods games. Naturally, the observed difference is more prominent when Q is larger and of the same order as M.

Most public goods dilemmas lead to one and only one stable strategy when one of the limits of large number of communities or weak selection is considered, as was noted in sections 3.1 and 3.2 of the main text. Under the S and the TS, the only exceptions to that rule, there are some cases of bi-stability when the system is close to neutrality, under which none of the strategies fixates on the other. Overall, this means that in either of those limits, if the fixation of cooperators is favoured by selection, we can conclude that cooperation will necessarily be a stable strategy. In settings with strong selection and a finite number of communities, such as the ones explored in this section, we have not observed any outcome where mutual fixation occurs, thus suggesting that the previous conclusion might hold for more general regions of the parameter space.

Under the HD dilemma, the only commons dilemma studied here, cooperators fixate successfully when $V/K < (V/K)^c$. Therefore, from Fig C, we observe that higher values of intensity of selection lead to larger regions in which cooperation fixates. As noted before, cooperation always fixates when $Q = 2$, and therefore we haven't represented in the figure the value of $(V/K)^c$ for that case. Increasing the community size to $Q = 3, 4$ lowers the critical values of V/K, thus leading to smaller regions of fixation of cooperators. However, increasing it to $Q = 5$ and above leads to a change in the opposite direction, thus increasing the regions where cooperation fixates. This effect has been described and analysed in section 3.3 of the main text in the context of weak selection and a large number of communities, and it is valid when those limits are relaxed, as can be observed in Fig C.

References

[1] Karlin S, Taylor HM. A First Course in Stochastic Processes. 2nd ed. New York, USA: Academic Press; 1975.

[2] Moran PAP. Random processes in genetics. Mathematical Proceedings of the Cambridge Philosophical Society. 1958;54(1):60-71.

[3] Nowak MA, Sasaki A, Taylor C, Fudenberg D. Emergence of cooperation and evolutionary stability in finite populations. Nature. 2004;428:646-50.

[4] Sample C, Allen B. The limits of weak selection and large population size in evolutionary game theory. Journal of Mathematical Biology. 2017 11;75(5):1285-317.
