# Supplementary material for: The rules of multiplayer cooperation in networks of communities
Source: PLoS Comput Biol. 2024 Aug 19;20(8):e1012388. doi: 10.1371/journal.pcbi.1012388 (PMC11361752; doi:10.1371/journal.pcbi.1012388)
Supplement: Attachment — Submitted filename: response to reviewers.pdf [file pcbi.1012388.s002.pdf]

Response to Reviewers at PLoS Computational Biology

July 30, 2024

We would like to thank the reviewers for carefully reading our manuscript and making numerous excellent suggestions that allowed us to improve it significantly. Below we answer each comment to the best of our ability. Alterations and additions to the original manuscript and supplementary material were included in blue text in the newly submitted documents.

Referee 1

1. "The paper includes a large number of parameters — it might be useful to include a table (in the main text of Supporting Information) that summarises these parameters, including relevant information such as their ranges, etc."

   Response: This is a good point. We have now added a new section coming first in the supplementary material, where a table with the free parameters and their meaning are described. We include text support to that table where we describe which values of the parameters are considered in each part of the results section of the main text, and which parameters are studied for all possible values. Furthermore, we have added text to sections 2 and 3 (previous sections 1 and 2) of the supplementary material clarifying the relation between the free parameters whose limits were considered.

2. "The paper focuses on cooperation/social dilemma settings. It seems meta-population evolutionary game methods have been applied to several other contexts, e.g. AI governance and safety (see e.g. "Trust AI Regulation? Discerning users are vital to build trust and effective AI regulation." arXiv preprint arXiv:2403.09510 (2024), and "Both eyes open: Vigilant Incentives help Auditors improve AI Safety." Journal of Physics: Complexity (2024).), environmental monitoring ("Paradigm shifts and the interplay between state, business and civil sectors." Royal Society open science 3.12 (2016): 160753.) and healthcare investment ("Toward Understanding the Interplay between Public and Private Healthcare Providers and Patients: An Agent-based Simulation Approach." EAI Endorsed Transactions on Industrial Networks and Intelligent Systems 7.24 (2020): 166668.). I think the developed methods in the current paper can be very useful to study behavioural evolution in these emerging contexts."

   Response: This is right, and even though there was a bit of discussion on other types of social dilemmas, namely those focused on consumption of pre-existing commons, there wasn't much discussion on realistic settings analysed using evolutionary games. We added a few lines in the discussion section mentioning that the framework can be applied to current topics where behavioural dynamics are important and we cite four recent papers where this is discussed.

Referee 2

1. "The model itself is fragile and the weaknesses and border cases are not properly addressed. Namely: the probability that an individual interacts with some other group is 1/(h+d) where d is the degree. This choice seems robust, but changing the probability to 1/h*1/d (with probability 1/h it "misses" his group and then chooses randomly) gives a very different dynamic. (more similar to the moran process). Therefore some strong justification is needed and some clarification about the limits of the model will also be helpful."

   Response: As suggested by the reviewer, alternative movement models could have been used.

   Regarding the motivation of the movement model used in our work, we chose a model governed by a single parameter yet allowing for different movement propensities governed by the opportunities available, reflecting basic characteristics of local limited mobility present in animal populations based on territorial behaviour [1, 2, 3] as well as human social systems. Following this movement probability, an individual (I_n) with more neighbouring options to move (d_n) has higher total probability of moving ($1 - h/(h + d_n)$), but lower probability of moving to one of the neighbouring places ($1/(h + d_n)$), treating the home place as h times as likely as any particular neighbouring place. This is a middle ground option between the following two extremes: 1) modelling all individuals with the same probability of staying in their home node; or 2) modelling all individuals with the same probability of moving to one of the neighbouring nodes. To clarify this point, we have motivated the use of this model by adding a couple of points to the Model section.

   Alternative models could have been considered, in particular the one proposed by the reviewer is an interesting proposition which fixes the probability of individuals staying at home at one single value, regardless of the degree of their home node. This would lead to differences under low values of home fidelity h if we had a very heterogeneous graph. For large values of h, as the ones considered throughout our work, even if we consider heterogeneous graphs, there would be no differences to the core results, i.e. the fixation probabilities calculated and the rules of cooperation derived from them. However, this choice of model would affect other aspects of the dynamics which we did not focus on, such as the rates at which between-community events occurred. To clarify these aspects, we have added a new paragraph with some points of discussion about considering alternative movement models to section 2.1 (previous section 1.1) of the Supplementary Information, when the fixation probabilities are derived. These comments are referred to in the Model section when the movement model is introduced, and in the Results section when the general fixation probabilities are presented.

2. "A clearer explanation for Figure 1 is desirable: it seems that in the next step, the individual can move and then stay in a different community. (It's not true, but from the first reading it seems correct and this process seems more interesting.)"

   Response: The reviewer raises a solid criticism, which we addressed by expanding on the description of the figure and explaining the following:

   "At each time step, individuals initiate their movement from their home node, and can either remain there or move to any of the adjacent nodes, returning to their home

node prior to the next time step. In this figure, we represent the resulting probability distribution for the community in the centre of the network."

This should make it clear that individuals do not move from node to node, but instead are always based in the same home node, and return to it after an interaction in one of the adjacent nodes.

3. "The fitness of an individual (eq 3) makes me a bit uncomfortable. The model should simulate a discrete process. That means the payoff should be the realization of the interaction within the community (with current members), not the expectation over all possible configurations. Here, it might be desirable that everyone having the payoff computed from the original community. (The current choice looks more general, but it's not)"

   "Again, in eq (5), there could be more straightforward (with weight 1/(h+d) we spread into different communities). This again looks more general than it is and makes the model unnecessarily complicated."

   Response:

   We consider these two points to be strongly related. In our model, we consider both the fitness and replacement weights of individuals to be computed assuming a large sample of random interactions within their environment, as has been widely done both in pairwise games [4, 5, 6], and multiplayer games [7, 8, 9]. This is a typical approach within mathematical and some computational descriptions of evolutionary games on graphs.

   Alternatively, these could have been calculated using different sampling assumptions. From our understanding of the reviewers comments, they propose an alternative way of computing fitness and replacement weights based on the individuals' last interacting group. This is a different way of considering the impact of interactions on the evolutionary process, as it considers fitness to simply be the most recent payoff of individuals in a stochastic process. This alternative has been considered in other works such as [10, 11], where computational simulation models are developed instead of mathematical ones, and this sampling assumption is used in order to avoid excessive computation time in calculating the long term fitness of individuals in the population.

   When that's the way considered to calculate fitness and replacement weights, there might be other effects emerging if the sampling used to calculate both quantities is correlated. It was shown in [12], that if interaction partners and competitors are sampled together, cooperation is increasingly more difficult to evolve. However, we follow the alternative mean-field sampling assumptions that fitness is obtained from the accumulated payoffs received on all possible group compositions and replacement weights are obtained from the fraction of time spent between any two individuals.

   Furthermore, we would like to note that the proposed way of computing fitness and replacement weights is general enough to allow different types of movement and spatial models – see a similar one in [7, 8] and other alternatives in [9, 13, 14]. Due to its general character, the general model seems indeed complicated, as the reviewer suggests. In the present paper, we did not intend to introduce a completely new simple model to study community structure, but instead we proposed to study the dynamics of this previously existing multiplayer interaction model in the limit of large home fidelity. What we successfully achieve in our work is to derive the simpler

evolutionary dynamics and the rules of cooperation obtained in the limit of high home fidelity. We describe in the manuscript that the complex model is not only simplified under large home fidelity, but also shows a lot of similarities to traditional models of metapopulation dynamics.

Following the reviewer's comment, we acknowledge the possibility of using different sampling assumptions to calculate fitness and replacement weights, and as a result we add some discussion points to the manuscript when the model is introduced.

We thank again referees 1 and 2 for their comments and hope everything is addressed accordingly.

References

[1] Ketterson E, Nolan Jr V. Site attachment and site fidelity in migratory birds: experimental evidence from the field and analogies from neurobiology. In: Bird migration: physiology and ecophysiology. Springer; 1990. p. 117-29.

[2] Woodroffe R, Ginsberg JR, Macdonald DW. The African wild dog: status survey and conservation action plan. Gland, Switzerland: IUCN; 1997.

[3] Woodroffe R, Ginsberg JR. Conserving the African wild dog Lycaon pictus. I. Diagnosing and treating causes of decline. Oryx. 1999;33(2):132-42.

[4] Ohtsuki H, Hauert C, Lieberman E, Nowak MA. A simple rule for the evolution of cooperation on graphs and social networks. Nature. 2006 5;441(7092):502-5.

[5] Santos FC, Pacheco JM. Scale-free networks provide a unifying framework for the emergence of cooperation. Physical Review Letters. 2005 8;95(9).

[6] Santos FC, Santos MD, Pacheco JM. Social diversity promotes the emergence of cooperation in public goods games. Nature. 2008 7;454(7201):213-6.

[7] Pattni K, Broom M, Rychtář J, Silvers LJ. Evolutionary graph theory revisited: When is an evolutionary process equivalent to the Moran process? Proceedings of the Royal Society A: Mathematical, Physical and Engineering Sciences. 2015;471(2182).

[8] Pattni K, Broom M, Rychtář J. Evolutionary dynamics and the evolution of multiplayer cooperation in a subdivided population. Journal of Theoretical Biology. 2017 9;429:105-15.

[9] Pires DL, Erovenko IV, Broom M. Network topology and movement cost, not updating mechanism, determine the evolution of cooperation in mobile structured populations. PLoS ONE. 2023;18(8):e0289366.

[10] Schimit PHT, Pattni K, Broom M. Dynamics of multi-player games on complex networks using territorial interactions. Physical Review E. 2019;99(3).

[11] Schimit PHT, Pereira FH, Broom M. Good predictors for the fixation probability on complex networks of multi-player games using territorial interactions. Ecological Complexity. 2022 10;51:101017. Available from: `https://linkinghub.elsevier.com/retrieve/pii/S1476945X2200037X`.

[12] Hauert C, Miekisz J. Effects of sampling interaction partners and competitors in evolutionary games. Physical Review E. 2018;98(5):052301.

[13] Pattni K, Broom M, Rychtář J. Evolving multiplayer networks: Modelling the evolution of cooperation in a mobile population. Discrete and Continuous Dynamical Systems - Series B. 2018 7;23(5):1975-2004.

[14] Erovenko IV, Bauer J, Broom M, Pattni K, Rychtář J. The effect of network topology on optimal exploration strategies and the evolution of cooperation in a mobile population. Proceedings of the Royal Society A: Mathematical, Physical and Engineering Sciences. 2019;475(2230).
